# The Epidemiology of Hip and Knee Primary and Revision Arthroplasties during the COVID-19 Pandemic

**DOI:** 10.3390/healthcare9050519

**Published:** 2021-04-29

**Authors:** Krystian Kazubski, Łukasz Tomczyk, Bartosz Kopczyński, Piotr Morasiewicz

**Affiliations:** 1 Department of Orthopaedic and Trauma Surgery, University Hospital in Opole, Institute of Medical Sciences, University of Opole, al. Witosa 26, 45-401 Opole, Poland; dockk@o2.pl (K.K.); b.k.kopczynski@gmail.com (B.K.); 2 Department of Food Safety and Quality Management, Poznan University of Life Sciences, Wojska Polskiego 28, 60-637 Poznan, Poland; tomczyk@up.poznan.pl

**Keywords:** knee arthroplasties, hip arthroplasties, knee revision, hip revision, Covid-19, epidemiology, lock-down, pandemic, SARS-COV-2

## Abstract

Background: The purpose of this study was to provide a comprehensive assessment of the impact of the COVID-19 pandemic on the epidemiology of primary and revision arthroplasties of the hip and knee joint. Methods: This study compared the data on knee and hip arthroplasty procedures from 2 hospitals (primary and revision) conducted in two periods: the period of the COVID-19 pandemic in Poland (from 4 March 2020 to 15 October 2020) and the corresponding period prior to the pandemic (from 4 March 2019 to 15 October 2019). We compared the epidemiological data, demographic data, and hospital stay duration data from these two periods. Results: Our analysis demonstrated that the total number of hip arthroplasties conducted in 2020 decreased by 26% in comparison with 2019. In the case of knee arthroplasties, the total number of procedures in the evaluated period in 2020 decreased by 44%. Our study also showed that the mean time of hospital stay for orthopedic patients following hip or knee arthroplasty was 22.87% shorter. The female-to-male patient ratio decreased between the analyzed periods, and this was 22.96% lower during the pandemic. Conclusion: The COVID-19 pandemic in these two hospitals in Poland led to reduced numbers of hip and knee replacement procedures, shorter hospital stays, and a decreased female-to-male patient ratio. The mean age of patients undergoing hip or knee arthroplasty remained unchanged during the national lockdown with respect to the pre-pandemic figure.

## 1. Introduction

The most common indication for primary total hip or knee replacement is advanced osteoarthritis, with associated pain, problems with daily functioning, lower quality of life, and limited range of motion [1]. The indications for revision arthroplasty are loosening of the implant after a primary arthroplasty, periprosthetic fracture with loosening of the implant, and infection following the primary arthroplasty [2,3,4,5]. In the United States a total of 1–2 million hip and knee arthroplasties are performed each year [1,2], which has led to an estimated proportion of the population of over 50-year-olds who have undergone a hip or knee replacement procedure to be 2.34% and 4.55%, respectively [1].

The COVID-19 pandemic resulted in global changes in the functioning of healthcare facilities in 2020 [2,3,4,5,6,7,8,9,10,11,12,13,14,15,16,17,18,19]. The pandemic substantially limited public access to medical specialists; it also affected workflow routines and patient admission criteria in orthopedic wards [2,3,4,5,6,7,8,9,10,11,12,13,14,15,16,17,18,19]. Some healthcare professionals contracted SARS-CoV-2 and, consequently, had to undergo quarantine, while others were delegated to the care of COVID-19 patients, which affected the number of patients hospitalized at orthopedic wards [2,3]. During the period when the number of COVID-19 cases soared, elective procedures in some orthopedic wards were restricted [3,4,5,10] or completely halted [2,3,4,5]. Some patients, particularly those with comorbidities who had been qualified to undergo arthroplasty postponed their surgery for fear of contracting a COVID-19 infection. Meanwhile, there have been recommendations to resume elective hip and knee arthroplasties [6,7,8,11].

The important issue of the effect of the COVID-19 pandemic on the epidemiology of primary and revision hip and knee arthroplasties has been neither fully investigated nor understood. The available literature reports have estimated the effect of the COVID-19 pandemic on reduction in the number of hip and knee replacement surgeries in the United States, European centers where elective arthroplasties and revision arthroplasties are performed, and hip and knee arthroplasties conducted in Italy [2,3,4,5,10]. There have been no studies analyzing in detail the impact of COVID-19 pandemics on the number of primary and revision hip and knee arthroplasties. There were also no studies analyzing in detail the impact of COVID-19 pandemics on the mean age of patients, mean hospitalization time and female to male ratio in patients after primary and revision hip and knee arthroplasties.

The epidemiology of hip and knee arthroplasties appears to have changed during the COVID- 19 pandemic. Most of the differences in the time of stay in hospital for patients and the total number of operations are not due to the COVID- 19 pandemic, but because of the health procedures followed, and sometimes because of patients’ fear of long stays in health facilities and the chances of them being infected with COVID19.

The purpose of this study was to provide a comprehensive assessment of the impact of the COVID-19 pandemic on the epidemiology of primary and revision arthroplasties of the hip and knee joint in 2 hospitals in Poland.

## 2. Methods

### 2.1. Study Design and Patients

This study analyzed the epidemiology of primary and revision hip and knee arthroplasty procedures conducted at two orthopedic centers in Poland dealing with this type of procedure, as well as medical rehabilitation. The medical database of all data for all patients at our hospital was analyzed to collect data for this study. Our analysis included a comparison of data collected during the period of the COVID-19 pandemic in Poland (between 4 March 2020 and 15 October 2020) with those collected during the corresponding pre-pandemic period (between 4 March 2019 and 15 October 2019). The study was approved by the local review board.

### 2.2. Evaluation of Epidemiological Parameters

The inclusion criterion was a history of one of the following procedures, i.e., total hip arthroplasty, total knee arthroplasty, revision arthroplasty of the hip, or revision arthroplasty of the knee, during one of the evaluated periods (i.e., between 4 March 2019 and 15 October 2019 or between 4 March 2020 and 15 October 2020). The collected data was analyzed for the total number of hip and knee arthroplasties; total number of women who underwent a hip replacement surgery (primary or revision); total number of men who underwent a hip replacement surgery (primary or revision); total number of women who underwent a knee replacement surgery (primary or revision); and total number of men who underwent a knee replacement surgery (primary or revision). In addition, we analyzed the mean age of patients (male and female separately) who underwent a knee replacement surgery (primary or revision); mean age of patients (male and female separately) who underwent a hip replacement surgery (primary or revision); mean age of these orthopedic patients, irrespective of their sex, who underwent a hip replacement surgery (primary or revision); and mean age of patients, irrespective of their sex, who underwent a knee replacement surgery (primary or revision). Moreover, we evaluated the mean duration of hospital stay following (primary or revision) hip replacement surgery (for all patients, males, and females); mean duration of hospital stay following (primary or revision) knee replacement surgery (for all patients, males, and females); and the ratio of female to male patients.

All the evaluated data from the period of the COVID-19 pandemic in Poland (from 4 March 2020 to 15 October 2020) were compared with the corresponding data from the period prior to the COVID-19 pandemic in Poland (from 4 March 2019 to 15 October 2019).

### 2.3. Statistical Analysis

The obtained data were statistically analyzed using the Statistica 13.1 software. Pearson’s chi-square test, t-student test, Mann-Whitney U test, correlation analysis and one-way ANOVA were used to compare the variables. Pearson’s chi-square test was used to assess the relationship between the frequency distribution of responses in one variable with respect to the other variable. Student’s t-test was used to compare the continuous variables for two groups (during and before the pandemic). The level of significance was set at α = 0.05.

## 3. Results

All results are presented in Table 1.

**Figure 1:**
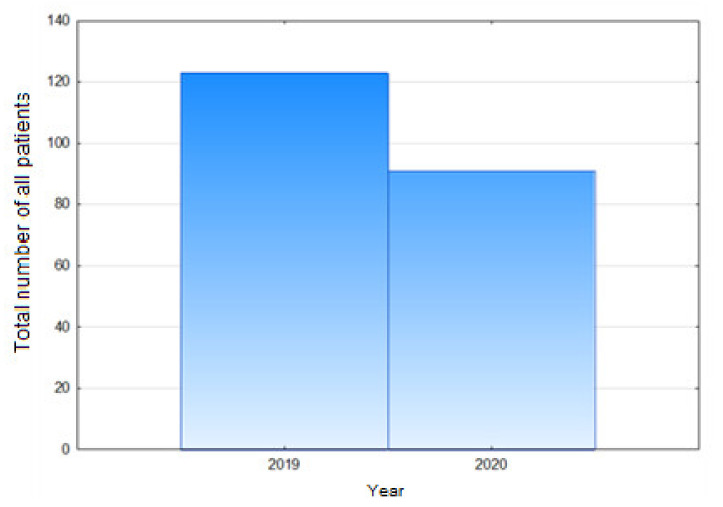
Total number of all patients—hip endoprosthesis.

Our analysis demonstrated that the total number of hip arthroplasties conducted in 2020 (i.e., in the evaluated period during the pandemic) decreased by 26% in comparison with the number of hip arthroplasties conducted in the corresponding period in 2019. This difference was statistically significant (Figure 1), (*p* = 0.0456).

**Figure 2:**
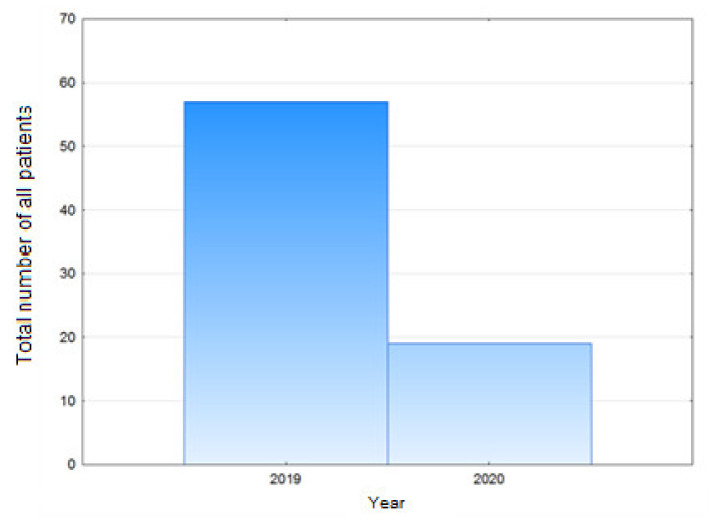
Total number of all patients—knee endoprosthesis.

In the case of knee arthroplasties, the total number of procedures in the evaluated period in 2020 decreased by 43.86% in comparison with the number of knee arthroplasties conducted in the corresponding period in 2019. This difference was statistically significant (Figure 2), (*p* = 0.0024).

The total number of hip revision conducted in 2020 decreased by 30%. The total number of knee revision conducted in 2020 increased by 100%, but this difference was not statistically significant (*p* = 0.24723).

**Figure 3:**
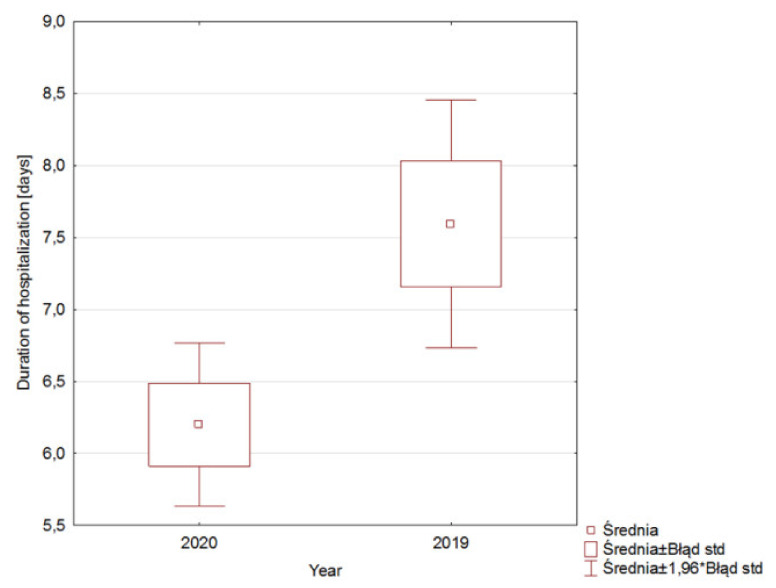
Duration of hospitalization.

Our study also showed that the mean time of hospital stay for orthopedic patients following hip or knee arthroplasty was 22.87% shorter (from 7.52 days to 5.8 days). This difference was statistically significant (Figure 3), (*p* = 0.012). The mean time of hospital stay for patients following hip revision was 34.05% longer (from 9.25 days to 12.4 days). However, this difference was not statistically significant (*p* = 0.53931). The mean time of hospital stay for patients following knee revision was 43.75 % shorter (from 8 days to 4.5 days). This difference was not statistically significant (*p* = 0.35007).

The mean age of orthopedic patients undergoing the evaluated procedures during the pandemic was lower (from 70.19 years in 2019 to 68.13 years in 2020). However, this difference was not significant (*p* = 0.1557). The mean age of patients following hip revision during the pandemic increased (from 74.75 years in 2019 to 77 years in 2020), but this difference was not significant. The mean age of patients following knee revision during the pandemic increased (from 62 years in 2019 to 70.25 years in 2020). However, this difference was not significant.

**Figure 4:**
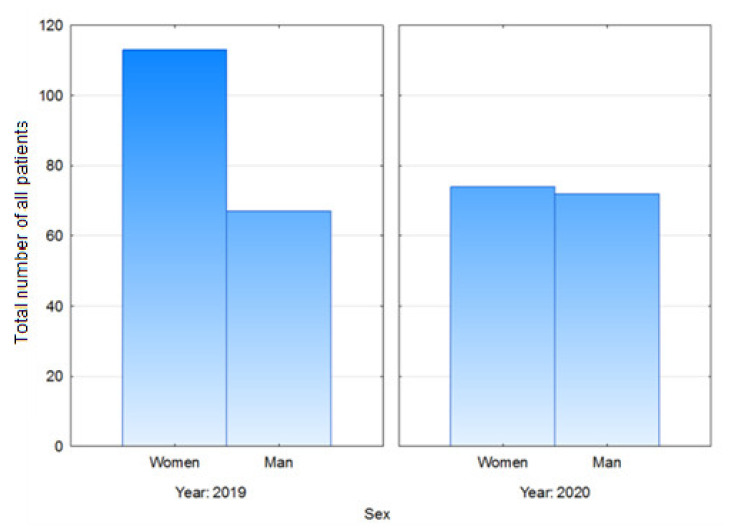
Female to male ratio.

The female to male patient ratio decreased between the analyzed periods, and was 22.96% lower during the pandemic. This difference was statistically significant (Figure 4), (*p* = 0.02813).

## 4. Discussion

Due to the large number of patients with hip and knee arthropathy worldwide and, consequently, the total number of hip and knee replacement operations globally [1,2], it is important to assess the epidemiology of hip and knee arthroplasties during the COVID-19 pandemic.

Previous studies assessed the effects of the COVID-19 pandemic on lowering the numbers of hip and knee arthroplasties conducted in the United States, European centers conducting primary and revision elective arthroplasties, and hip and knee arthroplasties conducted in Italy [2,3,4,5,10]. There have been no studies analyzing in detail the effect of national lockdown measures on the epidemiology of primary and revision arthroplasties of the hip and knee in Poland and no studies analyzing in detail the impact of COVID-19 pandemics on the number of primary and revision hip and knee arthroplasties. There were also no studies analyzing in detail the impact of COVID-19 pandemics on the mean age of patients, mean hospitalization time and female to male ratio, in patients after primary and revision hip and knee arthroplasties, which is a significant problem for analysis.

The COVID-19 pandemic has considerably altered the way the entire healthcare systems operate, including the way orthopedic wards function [2,3,4,5,6,7,8,9,10,11,12,13,14,15,16,17,18,19]. Doctors and nurses from orthopedic wards found themselves in a completely new situation. Some of the medical personnel contracted SARS-CoV-2 and had to undergo quarantine, while some were delegated to work with COVID-19 patients [2,3]. In order to curb the spread of COVID-19, hospital managers decided to restrict or completely halt elective orthopedic surgeries [2,3,4,5,10]. Additionally, the patients themselves, due to fears of COVID-19 infection, largely canceled or postponed their elective orthopedic procedures. All of the above factors had an impact on the epidemiology of these procedures, including arthroplasties. Meanwhile, there were recommendations to resume elective hip and knee replacement surgeries [6,7,8,11].

Bedard estimated a 75–100% drop in the number of hip and knee arthroplasties during the pandemic in the United States [2]. There were reports of primary arthroplasties being conducted in only 5.9–31.9% of qualified centers, with revision arthroplasties conducted in only 11.8–18.8% during the COVID-19 pandemic [3,4,5]. In Italy, the COVID-19 pandemic saw a 76.5% reduction in the number of arthroplasties [10]. Our study showed the total number of hip and knee arthroplasties during the pandemic to be lower by 26% and 44%, respectively. The total number of hip revisions conducted in 2020 decreased by 30%, but the total number of knee revisions conducted in 2020 increased by 100%. The COVID-19 pandemic, in the two hospitals in Poland analyzed by us, has not led to the number of hip and knee primary and revision arthroplasties conducted in our orthopedic ward dropping as dramatically as it has in other European centers and centers in the United States [2,3,4,5,10]. This may be explained by the fact that, in our orthopedic ward in Poland, patients must wait for a long time in order to have their elective hip or knee replacement surgery (which makes them more determined not to miss their surgery date) and by the lack of pandemic-related restrictions on arthroplasties performed at our two orthopedic wards.

There have been no studies evaluating the impact of the COVID-19 pandemic on the age of patients undergoing hip or knee replacement surgeries. Our study showed no significant effect of the national lockdown in the mean age of patients who underwent hip or knee primary and revision arthroplasty. The observed lack of a decrease in the mean patient age seems to be associated with patients in our orthopedic ward being highly determined to undergo their elective orthopedic procedures.

There have been no studies assessing the effect of lockdown measures on the mean duration of hospitalization for hip or knee replacement surgery. The data from our center showed the mean duration of hospital stay after primary and revision knee surgery during the pandemic to be shorter than that prior to the pandemic. This was due to several factors: doctors were more eager to expeditiously discharge orthopedic patients from the ward to minimize the risk of COVID-19 infection; in-hospital rehabilitation facilities were more accessible than at other times, which accelerated the patients’ postoperative recovery; and the patients themselves were more eager to be discharged for fear of COVID-19 infection.

There has been a scarcity of data on the effect of the COVID-19 pandemic on the ratio of females to males among those patients who undergo hip or knee replacement. Some authors report higher numbers of hip and knee replacements being conducted in women than in men [1]. Our study showed the female-to-male ratio during the national lockdown to be lower by 22.96% than that before the pandemic. This may have been due to female patients being more afraid of contracting COVID-19 than male patients.

One limitation of our study is the fact that the analyzed epidemiological data came from two centers. In the future, we are planning a multi-center study to broaden the scope of evaluated data. In this light, due to the fluid nature of the COVID-19 pandemic, findings reported may not be relevant for all countries going forward.

## 5. Conclusions

Our study showed a significant impact of the COVID-19 pandemic on the epidemiology of hip and knee arthroplasties.

The COVID-19 pandemic in our two orthopedic wards in Poland led to reduced numbers of hip and knee primary replacement procedures, reduced numbers of hip revision, increased number of knee revision, shorter hospital stays, and decreased female-to-male patient ratio.

The mean age of patients undergoing hip or knee arthroplasty remained unchanged during the national lockdown with respect to the pre-pandemic figure.

The COVID-19 pandemic in our two orthopedic wards in Poland has not led to the number of hip and knee arthroplasties conducted to drop as dramatically as it has in other European centers and centers the United States.

## Figures and Tables

**Table 1:** Epidemiological characteristics of patients who underwent hip and knee arthroplasty.

Variable	2020 Pandemic	2019 No Pandemic	Differences between 2020 vs. 2019	*p*	Pearson Correlation Coefficient
Total number of patients—hip endoprosthesis	91	123	−26%	0,0456	0,59
women	55	74	−25,68%	0,0243	0,53
men	36	49	−26,53%		
Total number of all patients—knee endoprosthesis	32	57	−43,86%	0,0024	0,69
women	19	39	−51,28%	0,00033	0,71
men	13	18	−27,77%		
Total number of hip revision	14	20	−30,00%		
Total number of knee revision	4	2	100,00%		
mean age of all patients	68,13	70,19	−2,93%	0,1557	0,12
mean age of women -hip endoprosthesis	74,36	75,66	−1,72%	0,8512	0,09
mean age of men—hip endoprosthesis	66,7	68,34	−2,40%		
mean age of women -knee endoprosthesis	68,32	70,12	−2,57%	0,6827	0,19
mean age of men—knee endoprosthesis	63,15	66,7	−5,32%		
mean age of hip revision					
mean age of knee revision					
medium duration of hospitalization (days)	5,8	7,52	−22,87%	0,012	0,64
medium duration of women hospitalization—hip endoprosthesis (days)	7,03	7,08	−0,70%	0,01714	0,58
medium duration of men hospitalization—hip endoprosthesis (days)	6	9,8	−38,77%		
medium duration of women hospitalization—knee endoprosthesis (days)	4,5	5,8	−22,41%	0,023	0,45
medium duration of men hospitalization—knee endoprosthesis (days)	5,8	7,4	21,62%		
medium duration of women hospitalization—hip revision (days)					
medium duration of women hospitalization—knee revision (days)					
female to male ratio	1,51	1,96	−22,96%	0,02813	0,57

## Data Availability

Not applicable.
